# Endophthalmitis patients in Makassar City: molecular identification of pathogenic fungal profile

**DOI:** 10.1186/s12879-024-10209-2

**Published:** 2024-11-19

**Authors:** Willy Wirawan Guslianto, Yunialthy Dwia Pertiwi, Mochammad Hatta, Lisa Tenriesa, Ririn Nislawati, Fadhilah Syamsuri, Muhammad Nasrum Massi, Firdaus Hamid

**Affiliations:** 1 Department of Microbiology, Faculty of Medicine, Hasanuddin University, Perintis Kemerdekaan No.Km. 10, Tamalanrea Indah, Kec. Tamalanrea, Kota Makassar, Sulawesi Selatan, Makassar, 90245 Indonesia; 2 Department of Ophthalmology, Faculty of Medicine, Hasanuddin University, Makassar, Indonesia; 3 Microbiology Laboratory, Wahidin Sudirohusodo Hospital, Makassar, Indonesia

**Keywords:** Endophthalmitis, Fungal profile, Polymerase chain reaction, Culture-negative

## Abstract

**Background:**

Endophthalmitis is a severe inflammation of the internal ocular structures, usually caused by bacterial or fungal infections, and can lead to rapid, irreversible blindness. Fungal endophthalmitis (FE), primarily due to *Candida albicans* and *Aspergillus*, is less common than bacterial endophthalmitis but has shown an increase in prevalence over the past two decades. Diagnosing FE is challenging and often delayed due to the time-consuming nature of traditional culture methods. The timely initiation of targeted antifungal therapy based on the specific fungal pathogen identified by molecular method can improve patient outcomes and reduce the risk of vision loss. This study aims to determine the presence of pathogenic fungal infections in patients with endophthalmitis using molecular methods at Hasanuddin University Hospital Makassar.

**Methods:**

This cross-sectional observational study analyzed 83 intraocular fluid samples from patients with endophthalmitis at Hasanuddin University Hospital, Makassar, Indonesia. Samples were examined using microscopy, culture, and molecular methods, including polymerase chain reaction (PCR) and deoxyribonucleic acid (DNA) sequencing.

**Results:**

The study population comprised 49 males (59%) and 34 females (41%), with an average age of 45.85 years. The distribution of affected eyes was nearly equal, with 50.6% involving the right eye and 49.4% involving the left eye. Exogenous transmission, primarily related to external risk factors such as ocular trauma or surgical procedures, was identified as the most common mode of fungal transmission in this population (97.6%). No fungal elements were detected through microscopy or culture; however, PCR could identify 5 positive samples (6%); 3 were males and 2 were females; all have exogenous transmission, predominantly showing *Candida* species. Sequencing revealed *Candida parapsilosis*,* Lodderomyces beijingensis*, and *Trichophyton rubrum* among the findings.

**Conclusion:**

Cases of fungal endophthalmitis are rare but increasing, posing diagnostic challenges. Our study concludes that PCR is more effective than traditional culture methods in identifying fungal pathogens, with a predominance of *Candida species* identified in endophthalmitis. Molecular techniques like PCR offer rapid and accurate diagnosis, improving patient treatment outcomes by enabling earlier initiation of targeted antifungal therapy.

**Supplementary Information:**

The online version contains supplementary material available at 10.1186/s12879-024-10209-2.

## Introduction

Endophthalmitis is an inflammation of the internal ocular structures, specifically the vitreous and aqueous humor [1]. Endophthalmitis can cause irreversible blindness within hours or days of symptom onset. The term endophthalmitis is more closely associated with bacterial or fungal infections, while viral or parasitic infections are usually associated with uveitis [2]. Based on the mode of transmission of the fungus, endophthalmitis can be exogenous, involving the introduction of an infectious source from outside the eye (e.g. trauma or surgical complications), or endogenous, involving the transit of an infectious source into the eye via the bloodstream [1–4]. 

Fungal endophthalmitis (FE) is endophthalmitis caused by fungi, most commonly yeast or mold [3, 4], with *Candida albicans* as the most commonly found yeast and *Aspergillus* as the most common mold in fungal endophthalmitis, with the most common symptom being vision loss accompanied by signs of inflammation [1, 5–7]. Compared to Asia, reports of fungal endophthalmitis are less common in Europe and North America [5]. The prevalence of fungal endophthalmitis, both exogenously and endogenously, is lower than bacterial endophthalmitis [1], however, in the last 20 years, culture positive cases of fungal endophthalmitis have increased from 8.6–18.6%.^3^ Delayed diagnosis and early misdiagnosis occur in 16–63% of cases [3, 8]. This is because diagnosing the etiology of fungal endophthalmitis is difficult and time-consuming [1, 3]. 

Fungal endophthalmitis is diagnosed by comprehensive clinical examination and confirmed by microbiological culture [1, 3, 4]. Although intraocular fluid culture is the gold standard, it has low sensitivity and time-consuming compared to polymerase chain reaction (PCR) [1, 4–12]. PCR of common organisms such as *Candida*,* Fusarium* and *Aspergillus* presents a relatively fast method of detecting fungal species, even when culture is negative [1, 3, 4, 11, 12]. 

The aim of our study was to determine the presence of pathogenic fungal infections in patients with endophthalmitis by molecular methods at the Hasanuddin University Hospital Makassar.

## Methods

### Research Settings and Design

This observational study with a cross-sectional approach examined 83 intraocular fluid samples, examined at the Microbiology Laboratory of Hasanuddin University Hospital, Makassar, Indonesia, with the inclusion criteria of all intraocular fluids taken from patients with endophthalmitis treated at Hasanuddin University Hospital, and the patient or patient’s guardian agreed to take part in this study. The exclusion criteria for this study were if the intraocular fluid samples sent were damaged (such as delayed transport time or the transport container breaking) or contaminated with other substances. Intraocular fluid specimens were analyzed by microscopic examination, culture and molecular detection based on standard methods at the Microbiology Laboratory of Hasanuddin University Hospital Makassar. Samples were extracted with the gSYNC™ deoxyribonucleic acid (DNA) Extraction Kit, followed by conventional PCR assays, and sequenced using the Automated DNA Sequencer ABI PRISM 377 (Perkin Elmer Biosystem, USA). All subjects provided written informed consent for inclusion before they participated in the study. The study was reviewed and approved by the Institutional Review of the Faculty of Medicine, Hasanuddin University Ethics Board with number UH23080537.

### Microscopy and culture

Intraocular fluid samples were taken for microscopic examination and culture with examination procedures following Clinical Microbiology Laboratory of Hasanuddin University Hospital Makassar standard operating procedures. Gram staining was performed, and samples were inoculated onto Blood Agar plates for isolation and further identification.

### Molecular examination

For the amplification [13, 14], each PCR mixture contained 25.0 µL of Go Taq Master Mix; 2.0 µL of primer mix containing 1.0 µL of the ITS1 10 μm primer set, and 1.0 µL of the ITS4 10 μm primer set; 13.0 µL of nuclease free water; 10.0 µL of DNA sample; in a final volume of 50 µL.

PCR amplification was performed in a BioRad Thermal Cycler. Duplicate PCR reactions were performed for each sample and were performed using a standard protocol involving denaturation, annealing, and extension steps. Thermocycling parameters included an initial denaturation step at 94^o^C for 2 min, followed by 30 cycles of 94^o^C for 30 s, 55^o^C for 30 s, 72^o^C for 90 s. The final extension step was performed at 72^o^C for 10 min. After amplification, PCR products were analyzed through agarose gel electrophoresis on 2% agarose gels containing ethidium bromide; 5 µL of PCR product was analyzed by electrophoresis at 100 V for 30 min.

Positive PCR results samples will continue with sequencing examination using the Automated DNA Sequencer ABI PRISM 377 (Perkin Elmer Biosystem, USA). Cycle sequencing of template DNA was carried out using the BigDye^®^ Ready Reaction Mix kit (Perkin Elmer Biosystem, USA). Phylogenetic analysis was conducted on sequences obtained from the NCBI Nucleotide database using BLAST (Basic Local Alignment Search Tool) and the Phylogenetic Tree Viewer (PTV).

### Data collection

Intraocular fluid samples examined at the Microbiology Laboratory of Hasanuddin University from March 2023 to April 2024, which had a fungal culture examination, were analyzed by collecting demographic data in the form of age, gender, affected eye area, and mode of transmission of endophthalmitis.

### Data analysis

Descriptive statistics were utilized to summarize patient demographics, and outcomes. The mean and standard deviation were calculated for continuous variables, and frequency and percentage were calculated for categorical variables.

## Result

A total of 83 specimens met the inclusion criteria for this study, followed by microscopic, culture and molecular examination.

The average age of the study population was 45.85 years, with a standard deviation of 23.96 years, suggesting a broad age range. Notably, men made up the majority (59.0%). For the affected eye, 50.6% were the right eye and 49.4% were the left eye. The most common mode of transmission for endophthalmitis in this study is exogenous (97.6%). All demographic data can be seen in Table 1 [see Additional File 1].

**Table 1:** Demographic of patients with intraocular fluid specimens

Demographics (*N* = 83)	Frequency, *n* (%)
Age (year, mean ± SD)	45.85 ± 23.96
Gender	
Male Female	49 (59.0) 34 (41.0)
Eye Affected	
Right Eye Left Eye	42 (50.6) 41 (49.4)
Mode of transmission	
Exogenous (e.g., trauma, post operative) Endogenous (e.g., fungemia, hematogenous)	81 (97.6) 2 (2.4)

SD : Standard deviation

There were no yeasts found on microscopic examination, and there were no positive fungal cultures. The PCR examination found five (6.0%) positive samples and continued with sequencing. Microbiological findings can be seen in Table 2.

**Table 2:** Microbiological findings

Laboratory findings (*N* = 83)	Frequency, *n* (%)
Gram Staining	
Yeast No yeast found	– 83 (100)
Fungal Culture	
Positive Negative	– 83 (100)
Polymerase Chain Reaction (PCR)	
Positive Negative	5 (6.0) 78 (94.0)

With an average age of 64.80 years and a standard deviation of 9.26, the demographics for PCR-positive samples showed that the majority of the study’s participants were old. There were 60% females and 60% right-eyed people affected. All of the modes of transmission for fungal endophthalmitis in this study are exogenous (100%), with 80% post-operative and 20% post-traumatic.

From the sequencing results, it was found that *Candida sp.* was the most abundant fungus in this study. Other species found from the sequencing were *Candida parapsilosis*, *Lodderomyces beijingensis*, and *Trichophyton rubrum*. The molecular findings can be seen in Table 3.

**Table 3:** Molecular findings

No.	Sample Number	Gram Stain (Yeast) / Fungal Culture	GenBank	Accession No.	DNA sequencing
1.	W_7	Negative / Negative	*JX406280.1*	*JX406280*	*Candida parapsilosis*
2.	W_38	Negative / Negative	*JX406299.1*	*JX406299*	*Candida sp.*
3.	W_46	Negative / Negative	*JX406299.1*	*JX406299*	*Candida sp.*
4.	W_78	Negative / Negative	*NC_089974.1*	*NC_089974*	*Lodderomyces beijingensis*
5.	W_80	Negative / Negative	*OW983267.1*	*OW983267*	*Trichophyton rubrum*

**Fig. 1:**
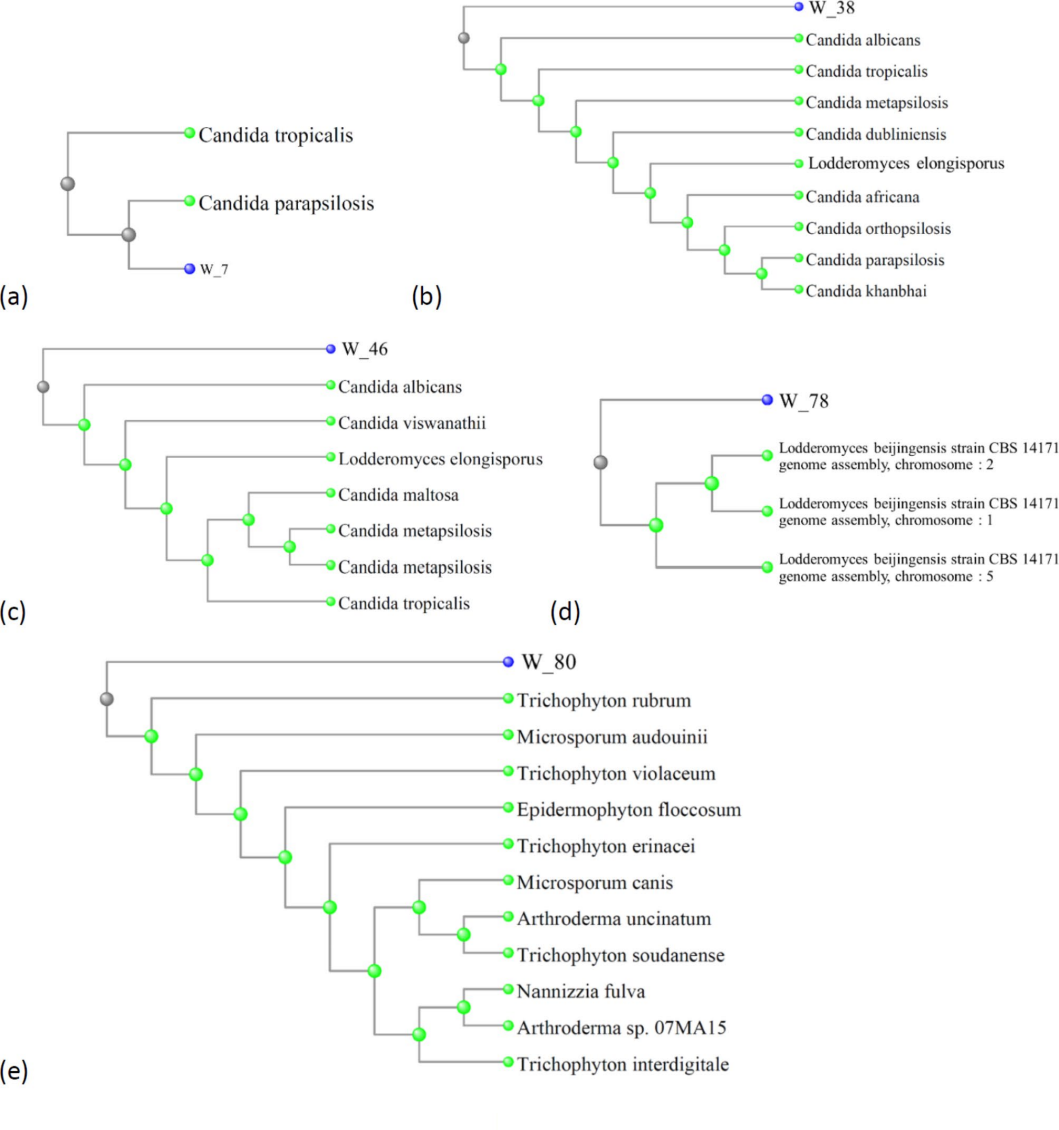
Phylogenetic tree based on sequencing for all yeast and mold. (**a**) Samples with code number W_7 have a 95.56% similarity with *Candida parapsilosis* with a genetic distance of 0–0.1. (**b**) Samples with code number W_38 have a 89.36% similarity with *Candida albicans* with a genetic distance of 0–0.04. (**c**) Samples with code number W_46 have a 93.33% similarity with *Candida albicans* with a genetic distance of 0–0.04. (**d**) Samples with code number W_78 have a 86.54% similarity with *Lodderomyces beijingensis* with a genetic distance of 0–0.07. (**e**) Samples with code number W_80 have a 85.42% similarity with *Trichophyton rubrum* with a genetic distance of 0–0.03

Understanding the evolutionary relationships and genetic similarities between various fungal species depends heavily on phylogenetic research. The phylogenetic tree of the fungi identified in this study can be seen in [Fig. 1].

## Discussion

Fungal endophthalmitis is a rare case that is less well diagnosed when compared with bacterial endophthalmitis. Molecular examination plays a crucial role in diagnosing fungal endophthalmitis due to several diagnostic challenges. One of the primary difficulties is that culture results are often negative, and the sensitivity of detecting fungi in intraocular fluid samples is low [1, 3–6, 8, 11]. In the early stages of infection, fungal endophthalmitis is frequently mistaken for bacterial infections, leading to inaccurate or delayed treatment, which can result in the progression to severe stages of the disease, increasing the risk of blindness. Given these challenges, PCR should be considered as a key diagnostic tool. The sensitivity of cultures of aqueous humor or vitreous had 17% in a retrospective research on eyes suspected of endophthalmitis or infectious uveitis, whereas PCR had 85%.^3^ PCR enables the rapid amplification of fungal DNA, allowing for the detection of minute amounts of fungal material, which is essential in cases where culture methods exhibit low sensitivity, particularly in intraocular fluid samples [4, 8]. DNA sequencing further enhances the accuracy by providing species-level identification of the pathogens, ensuring that treatment can be targeted appropriately. Compared to other molecular methods, PCR combined with sequencing offers a fast, more direct, and reliable approach to identifying fungal DNA, even in culture-negative cases or when bacterial presence complicates the diagnosis [3]. This makes PCR and DNA sequencing particularly advantageous in detecting fungal endophthalmitis, where conventional methods often fail to provide timely and accurate results, ensuring more precise treatment and improving patient outcomes. A targeted molecular approach is essential to differentiate fungal from bacterial infections early on, leading to timely and appropriate antifungal therapy.

This study highlights the crucial role of PCR in accurately diagnosing fungal endophthalmitis. While traditional methods like microscopy and fungal cultures failed to detect fungal pathogens in some cases [1, 3–6, 8, 11], PCR’s enhanced sensitivity brought to light the presence of fungal DNA in 5 (6.0%) of the total 83 samples. This underscores the importance of incorporating PCR into routine endophthalmitis diagnostic protocols to ensure timely and appropriate treatment, preventing potential vision loss.

Our study revealed an intriguing trend in the demographics of patients with PCR-positive fungal endophthalmitis. The average age of these individuals was 64.85 years, significantly higher than the overall study population’s average age of 45.85 years. This suggests an age-related susceptibility to fungal endophthalmitis, possibly due to weakened immune systems or underlying health conditions. This is similar to the case report presented by Hidalgo et al. [9], where the age of patients diagnosed with fungal endophthalmitis was generally over 60 years old. Additionally, our study found a higher proportion of males (59.0%) in all endophthalmitis cases, and among PCR-positive cases, there were more females (60.0%) compared to the overall male dominance (40.0%). When compared with the study conducted by Bhullar et al. [15]. and Belanger et al. [16], it was found that most cases of fungal endophthalmitis were dominated by men. While the reasons for this gender disparity remain unclear, it warrants further investigation to understand potential biological or behavioral factors at play.

Endophthalmitis can be classified into two modes of transmission: endogenous and exogenous. Rychener originally characterized exogenous endophthalmitis in 1933 and classified it as contiguous spread from an external ocular infection, penetrating trauma, and intraocular surgery [1]. All of the fungal endophthalmitis in our study was linked to exogenous transmission, in which the fungus enters the eye from an outside source. This finding aligns with previous research by Haseeb et al. [1] that up to 80% of instances of endophthalmitis are exogenous endophthalmitis, which is the most prevalent kind, and highlights the importance of preventive measures, such as proper hand hygiene and sterile surgical procedures, to minimize the risk of introducing fungal pathogens into the eye. Among all of the exogenous fungal endophthalmitis in our findings, 80% occurs post-operatively and 20% following eye trauma. In line with the studies of Saeedi et al. [4], Danielscu et al. [3], and Durand et al. [2], cases of postoperative endophthalmitis were reported more frequently when compared to traumatic or endogenous endophthalmitis. This suggests that surgical interventions and eye injuries create portals of entry for fungal pathogens, necessitating heightened vigilance and infection control measures in these settings.

Fungal findings in cases of endophthalmitis are very rare, as in our study only 5 (6.0%) out of 83 intraocular fluid samples were found to be fungal. This research is in line with studies Haseeb et al. [1], Danielscu et al. [3], and Belanger et al. [16], where the study has proven that cases of fungal endophthalmitis are rare. In a study conducted by Liu et al. [17] over 10 years (2011–2020) in northern China, of 524 patients with endophthalmitis cases, the rate of positive fungal cultures was only 12.21% (64 fungal isolates), whereas in our study, no fungal cultures were found to be positive. Danielscu et al. [3], in a retrospective study on eyes suspected of endophthalmitis or infectious uveitis, found that cultures of aqueous humor or vitreous had 17% sensitivity, while PCR had 85% (remaining relatively inexpensive). Our study found the molecular identification rate were higher than identification by conventional fungal culture, 6% and 0% respectively. Although microscopic examination and culture facilities are available in various hospitals and culture is still the gold standard for diagnosing intraocular infections, the culture sensitivity for intraocular specimens is very low [3, 5, 6, 8]. When there is a negative fungal culture, PCR is helpful and could be the method of choice in fungal detection [2, 3, 8, 10, 12]. PCR-based techniques have the potential to be far more sensitive than traditional cultures since they can detect very small quantities of DNA copies, which could allow for the establishment of an etiologic diagnosis in less time. Additionally, a PCR-based approach is highly helpful in cases of intraocular infections because to the vast variety of infectious microorganisms and the relatively small volume of sample that can be acquired at any one time [8, 9]. 

Sequencing of PCR-positive samples revealed *Candida sp.* as the most prevalent fungal pathogen in this study. This finding aligns with previous literature, such as Haseeb et al. [1], Bhullar et al. [15], and Belanger et al. [16], which demonstrated that *Candida albicans* was the most common fungal pathogen found in endophthalmitis, and Xu et al. [18], who demonstrated *Candida parapsilosis* can be the pathogen of postoperative endophthalmitis. Bhullar et al. [15] and Belanger et al. [16] studies also showed that *Candida parapsilosis* isolates were found to be fewer in fungal endophthalmitis cases. In the Bhullar et al. study [15], 3 (7.6%) out of 39 were *Candida parapsilosis* isolates when compared to *Candida albicans*, 23 (59.0%) out of 39, and in the Belanger et al. [19] study, 2 (9.1%) out of 22 were *Candida parapsilosis* isolates when compared to *Candida albicans*, 13 (59.1%) out of 22, whereas in our study, 60% of positive molecular examination results were obtained by *Candida sp.*, with 2 samples with *Candida albicans* genetic similarity and 1 sample with *Candida parapsilosis* genetic similarity. The finding of only 1 isolate of *Candida parapsilosis* in our study and in line with previous studies, indicates that this type of yeast is very rare in intraocular fluid in cases of endophthalmitis. Our study found one (20%) *Trichophyton rubrum* out of five fungal isolates. *Aspergillus sp.*,* Fusarium sp.*, and *Candida sp.* are the most frequent causes of exogenous fungal endophthalmitis [1, 4, 6, 7], however, there have been a few cases of fungal endophthalmitis caused by *Trichophyton sp.*, as reported by Supahiah et al. [20], Gaffar et al. [21] and Lin et al. [22] Despite this, *Trichophyton sp.* endophthalmitis cases are extremely rare. The presence of *T. rubrum* in intraocular fluid in our study suggests that conditions such as compromised immunity or prior ocular procedures may predispose individuals to infections by this otherwise superficial pathogen. This emphasizes the importance of molecular diagnostics in identifying unusual pathogens in endophthalmitis cases. One (20%) out of five of our fungal isolates also reveals *Lodderomyces beijingensis.* This microorganism has not been reported and is not generally considered a human pathogen [23, 24]. However, the detection of this microorganism in intraocular fluid suggests that certain host conditions, such as immune status, local ocular environment, or recent invasive procedures, may permit infections by typically non-pathogenic fungi. This rare finding highlights the need for further research involving larger sample sizes and diverse populations to understand the potential pathogenicity of *L. beijingensis* in ocular infections.

This research demonstrates that molecular-based examinations are helpful in detecting pathogens, especially fungi, if other tests yield negative results. It also highlights the need for caution in cases of endophthalmitis that can be caused by fungi, even though clinically they are similar to bacterial endophthalmitis, particularly in Makassar, Indonesia, where fungal endophthalmitis is thought to be rare, presents many diagnostic challenges, and there is still a lack of epidemiological data. The clinical implications of rapid and accurate PCR-based diagnosis are crucial, particularly in the context of molecular examination. PCR allows for the early detection of fungal pathogens, providing timely and precise results compared to traditional methods like culture, which can be slow or inconclusive. This rapid diagnosis enables clinicians to make informed treatment decisions more quickly, moving away from empirical therapies toward targeted antifungal treatment. As a result, patient outcomes improve, with faster recovery times and a reduced risk of complications, such as vision loss in endophthalmitis cases.

There are still limitations in this study, such as the fact that it is only a single-center study, the small amount of intraocular samples, and the small number of isolates found. Additionally, we recognize that molecular diagnostics, such as PCR, may not be readily accessible in many healthcare facilities, particularly in low-resource settings. The infrastructure and costs associated with PCR-based diagnostics can pose challenges to widespread implementation. Therefore, future efforts should focus on developing cost-effective, accessible diagnostic methods suitable for diverse clinical settings. Extensive epidemiological research, building upon the findings of this study, could enhance our understanding of the prevalence, risk factors, and clinical manifestations of fungal endophthalmitis across diverse geographic regions of Indonesia.

## Conclusion

Cases of fungal endophthalmitis are rare but increasing every year, which is a challenge because it is difficult to diagnose properly. Culture examination, which is the gold standard, often produces negative results. Our study concludes that the use of molecular techniques such as PCR has a greater advantage in fungal endophthalmitis diagnostics than routine microbiological examinations such as microscopic and culture. PCR helps in cases of negative fungal culture. *Candida sp.* were the most common fungi found in endophthalmitis cases in this study. With advances in molecular technology in the form of PCR, it can help to establish fungal etiology more rapidly and accurately, so that patients can be treated more precisely and better.

## Electronic supplementary material

Below is the link to the electronic supplementary material.

Supplementary Material 1

## Data Availability

Data is provided within the manuscript or Additional File 1.
